# Impact of Microplastic Contamination on Phosphorus Availability, Alkaline Phosphatase Activity, and Polymer Degradation in Soil

**DOI:** 10.3390/polym17121586

**Published:** 2025-06-06

**Authors:** Efsun Dindar

**Affiliations:** Department of Environmental Engineering, Faculty of Engineering, Bursa Uludag University, Bursa 16059, Turkey; efsun@uludag.edu.tr

**Keywords:** enzyme activity, microplastics, phosphorus, soil contamination

## Abstract

Microplastics (MPs) are emerging contaminants that can significantly impact soil nutrient dynamics, particularly phosphorus (P) cycling, which is critical for maintaining soil fertility and ecosystem productivity. However, limited information is available on how different microplastic types and concentrations specifically influence phosphorus dynamics and microbial enzyme activity in soils. Microplastic contamination may alter P cycling by directly supplying phosphorus or indirectly influencing microbial activity and enzyme function through changes in soil structure and aggregation. This study examined the short-term impacts of three widely used microplastic polymers—polyethylene (PE), polypropylene (PP), and polyethylene terephthalate (PET)—on soil phosphorus forms and alkaline phosphatase activity (APA), a key enzyme in phosphorus transformation. Incubation experiments were conducted at two concentrations (0.5% and 5%) over 30 and 60 days. The results indicated that the impact of microplastics on soil phosphorus dynamics varied according to both polymer type and contamination dose. Microplastics increased available phosphorus (AP) and APA levels compared to control soils, indicating a stimulatory effect on microbial processes. This may be due to the temporary accumulation of phosphorus on MP surfaces, which can stimulate phosphatase activity. Over time, however, both AP and APA levels declined, suggesting that degradation products released from MPs and organic matter may have altered the activity of the microbial communities responsible for P cycling. FTIR analysis revealed clear degradation of microplastics, with PET showing the most pronounced chemical transformation. PP exhibited moderate degradation, while PE demonstrated the highest resistance to environmental breakdown. These degradation processes likely released functional groups (e.g., carboxyl, carbonyl, hydroxyl) and low-molecular-weight compounds into the soil, modifying microbial processes and phosphorus chemistry. Particularly in PET-amended soils, these degradation products may have enhanced phosphate complexation or mobilization, contributing to higher levels of available phosphorus at the end of the incubation time. Understanding the polymer-specific and concentration-dependent effects of microplastics is critical for accurate ecological risk assessment in terrestrial ecosystems.

## 1. Introduction

Due to the massive manufacturing and consumption of plastic worldwide, microplastics are frequently introduced into soils and the ecosystem. Plastics undergo physical weathering and chemical breakdown in natural environments, progressively breaking down into tiny particles identified as microplastics that are less than 5 mm in size. Microplastics have been found to alter key nutrient cycling processes in soil.

Phosphorus is an essential macronutrient that plays a fundamental role in plant growth, microbial metabolism, and biogeochemical cycling. The soil P cycle is a complex interplay of physical, chemical, and biological processes that govern the availability of phosphorus in soil and influence plant growth.

Microplastics can affect how phosphorus is converted in the environment by altering microbial and enzyme activity or by supplying various phosphorus sources. Numerous studies have demonstrated that the presence of microplastics alters the environment’s organic and total phosphorus (TP) contents. According to Yu et al. [1], the total phosphorus content of the soil was considerably decreased by adding polystyrene (PS) and polyvinyl chloride (PVC). Conversely, Liu et al. [2] and Ren et al. [3] discovered that high concentrations of microplastics enhanced levels of dissolved organic phosphorus and phosphate, most likely as a result of increased enzymatic activity that accelerated the mineralization of organic phosphorus.

MPs may reduce P availability through adsorption and modification of soil structure. Their small particle size, large surface area, and functional groups allow them to bind phosphate ions, thus limiting their accessibility [4,5]. According to Zhang et al. [6], prolonged use of plastic film mulching reduced soil organic matter, which in turn affected microbial access to phosphorus. Additionally, MPs may affect soil water-holding capacity (WHC) and microaggregate stability [7], which can influence P mobility and uptake. MPs may directly impact water-holding capacity through hydrophobic surfaces. Higher WHC is thought to improve soil moisture conditions, which may enhance phosphatase activity by supporting microbial metabolism and contributing to the stabilization of enzymes within the soil matrix. According to Lozano et al. [8], well-watered soils have significantly higher phosphatase activity than dry soils. On the other hand, phosphatase activity is inhibited in well-watered soils and stimulated in dry soils when PET microplastics are added. Wan et al. [9] found that low MP concentrations (<0.5% *w*/*w*) can stimulate nutrient activity, whereas higher levels (1.0–3.0% *w*/*w*) exhibit toxic effects, reducing both AP and total P.

Enzyme activity in the soil is one example of a biological indicator that can show the health of the soil environment and the dynamic changes in ecosystems [10]. According to earlier research, adding MPs can either increase [11], reduce [12], or have no effect [13] on soil enzyme activity; these variations may be influenced by the type, dosage, and length of MP exposure as well as the characteristics of the soil. In order to transform organic phosphorus into inorganic phosphorus, phosphatase, an enzyme, catalyzes the hydrolysis of phosphate mono and phosphate diesters in the soil [14].

The various impacts of microplastics on soil phosphatase activity could be linked to the physicochemical parameters of the soil as well as the microplastics’ characteristics (such as type, shape, particle size, and concentration). Yi et al. [14] observed that phosphatase activity initially decreased and then increased in soils treated with membranous PE and fibrous PP, whereas microsphere PP led to persistent inhibition. Dong et al. [15] reported suppressed phosphatase activity in rice soils exposed to PS and polytetrafluoroethylene (PTFE) MPs under arsenic and amino acid stress. In contrast, Fei et al. [16] demonstrated that PE and PVC MPs enhanced phosphatase activity in acid soils.

Additionally, MPs have the ability to change the microbial populations in soil, especially the phosphate-solubilizing bacteria (PSB), which are in charge of turning insoluble inorganic phosphorus into phosphate ions that are accessible [17]. Increased AP levels have been attributed to enhanced microbial activity and PSB enrichment in several studies [18,19]. Yan et al. [20] observed increased PSB-associated Burkholderiaceae in soils treated with PE and plasticized PVC MPs. However, the effects are polymer- and soil-type-dependent; for example, in fertile paddy soils, unplasticized PVC decreased the AP content. Similarly, 0.5% phthalate-plasticized PVC exposure also promoted PSB abundance after 60 days [21]. These variations suggest that MP-induced changes in microbial structure and solubilizing capacity are key drivers of P dynamics [22].

Despite these findings, the interactions among MP polymer type, concentration, and exposure duration on phosphorus fractions and phosphatase activity remain unclear. Therefore, the purpose of this study was to evaluate the short-term effects of three commonly used microplastics—polyethylene (PE), polypropylene (PP), and polyethylene terephthalate (PET)—on alkaline phosphatase activity and soil phosphorus availability at varying concentrations (0.5% and 5%) and incubation times (30 and 60 days).

## 2. Materials and Methods

### 2.1. Materials

Soil samples were collected from the top 20 cm of an agricultural field located in Bursa-Balabancık village during the spring season (latitude, 40°15′55.1″ N; longitude, 28°47′07.55″ E). To ensure representativeness, a composite sample was prepared by thoroughly mixing subsamples collected from multiple points (*n* = 5) within the designated sampling area. Before being ground, the soil samples were allowed to air dry at ambient temperature. The experiment was carried out in the soil pollution laboratory of the university. The soil was contaminated with three different types of microplastics (PP, PE, and PET). Because of its many uses, polypropylene (PP) is a thermoplastic polymer that is widely utilized. PP is frequently utilized in the manufacturing of films, bottles, and containers, among other packaging materials. It may eventually deteriorate due to its sensitivity to ultraviolet (UV) rays. Another popular thermoplastic polymer that is well-known for its adaptability, toughness, and simplicity of processing is polyethylene (PE). PE is used to make mulching films, irrigation pipes, and greenhouse films for use in agriculture. Although polyethylene can be recycled, improper disposal or recycling may have negative environmental effects because of its persistence in the environment. A member of the polyester family, polyethylene terephthalate (PET) is a thermoplastic polymer. PET has superior carbon dioxide and oxygen barrier qualities. As such, it poses a serious risk to soil respiration. 

Prior to experimental use, the microplastics were reduced to the appropriate size by passing them through a <5 mm filter. Then, all microplastic particles were subjected to a purification process to eliminate potential surface contaminants and ensure analytical consistency. Initially, the particles were thoroughly washed with deionized water to remove loosely bound debris and dust. Subsequently, they were washed with ethanol to eliminate hydrophobic organic residues and possible manufacturing additives. For environmentally sourced microplastics, a mild alkaline treatment using a dilute sodium hydroxide (NaOH) solution was applied to facilitate the removal of persistent organic matter and surface biofilms. Following this step, mild detergents were also used where necessary to assist in removing proteinaceous residues. After each chemical treatment, the particles were thoroughly rinsed with deionized water to ensure the complete removal of any remaining reagents or surfactants. Finally, all microplastics were vacuum-dried at room temperature under clean and dust-free conditions [23].

The soil’s characteristics are shown in Table 1.

### 2.2. Incubation Experiment

In the experiment, 100 g of soil per pot was placed in glass pots to assess the impact of varying microplastic concentrations on soil phosphorus processes. Deionized water was added to the soil samples to bring their moisture content down to between 50% and 70% of their maximal water-holding capacity. Throughout the incubation phase, deionized water was frequently supplied to make up for any moisture lost through evaporation. The control group had soil devoid of microplastics.

In glass containers containing 100 g of dry soil, three different types of microplastics were added at two different weight percentages, 0.5% and 5%. The experiment included three treatment groups: Control: Soil with no added microplastics (MPs); Soil with 0.5% (*w*/*w*) MPs; Soil with 5% (*w*/*w*) MPs. The selected microplastic concentrations (0.5% and 5% *w*/*w*) were determined based on previous studies and environmental relevance [25]. The 0.5% concentration represents a realistic level of contamination that may occur in agricultural soils subjected to long-term plastic mulching, sewage sludge application, or wastewater irrigation [7,26]. The 5% concentration, on the other hand, was included to simulate a high-exposure scenario. This level was selected in reference to earlier studies investigating threshold effects and mechanistic responses of microplastics on soil biochemistry and microbial functions [16,27]. Together, these two concentrations allow for a comprehensive assessment of microplastic impacts on phosphorus cycling under both environmentally relevant and stress-induced conditions.

After adding the specified quantities of MPs to 100 g of dry weight soil, the soil was thoroughly mixed. The incubation experiments were conducted at 28 °C, which is considered an optimal temperature for the growth and metabolic activity of soil microorganisms. This temperature is commonly used in soil microcosm studies to support microbial processes under controlled laboratory conditions. For experimental purposes, three containers were utilized for each treatment at 30 and 60 days of incubation. The selected durations of 30 and 60 days were chosen to capture both short-term and intermediate effects of microplastic exposure on soil phosphorus dynamics and microbial activity. Similar incubation conditions have been applied in previous studies investigating the effects of microplastics on soil enzymatic activity and nutrient cycling [16,27,28]. The design of the experiment was completely randomized, and at the beginning of the incubation period, each treatment was carried out in triplicate.

### 2.3. Methods

#### 2.3.1. Soil Chemical Analyses

After 30 and 60 days of incubation, soil samples were collected, dried, and their alkaline phosphatase activity, total P, and available P were measured. A conductivity meter and a pH meter were used to measure the chemical characteristics of the soil. After shaking the soil with distilled water (1:5, *w*/*v*), the extracts were examined [29]. According to ISO 11261:1995 [30], the Kjeldahl digestion method was used to determine the total nitrogen concentration. Dichromate oxidation was used to measure the total amount of organic carbon [31]. The IC methods were used to determine nitrate (N) [32].

Total phosphorus was analyzed according to the SM 4500-P E method [33] and available phosphorus concentrations were determined using ICP-OES in accordance with the EPA 6010C method [34].

#### 2.3.2. Soil Enzyme Activity

The alkaline phosphatase (APA) enzymatic activity was measured using the Tabatabai [35] methodologies. A modified universal buffer (pH = 11), 0.025 M toluene, and p-nitrophenyl phosphate solutions were added to the soil in order to measure the alkaline phosphatase activities. After that, the samples from both analyses were incubated for one hour at 37 °C. Using a spectrophotometer (Hach Lange DR 5000; Hach Lange GmbH, Düsseldorf, Germany) set to 410 nm, the amount of released p-nitrophenol (PNP) was measured [36].

#### 2.3.3. Sample Preparation and Analysis of Microplastics in Soil

Soil samples were homogenized and weighed into glass beakers in quantities of 1 g, 5 g, and 10 g, with each amount prepared in triplicate. Subsequently, 20–50 mL of 10 M hydrogen peroxide solution (35%) was added to the samples, depending on their volume, to remove organic matter. Due to the high inorganic content of the soil samples, density separation was performed using a sodium chloride (NaCl) solution (density: 1.2 g/cm^3^). The NaCl-added samples were stirred with a magnetic stirrer and then allowed to settle for phase separation in a fume hood with their lids closed.

Once phase separation was achieved, the upper phase of the sample was carefully transferred to a clean beaker, and this procedure was repeated at least three times. After completing the separation steps, the collected upper phases were passed through a stainless-steel sieve and thoroughly washed with deionized water to remove residual NaCl. After that, the samples were dried at 40 °C in an oven [37].

**Figure 1:**
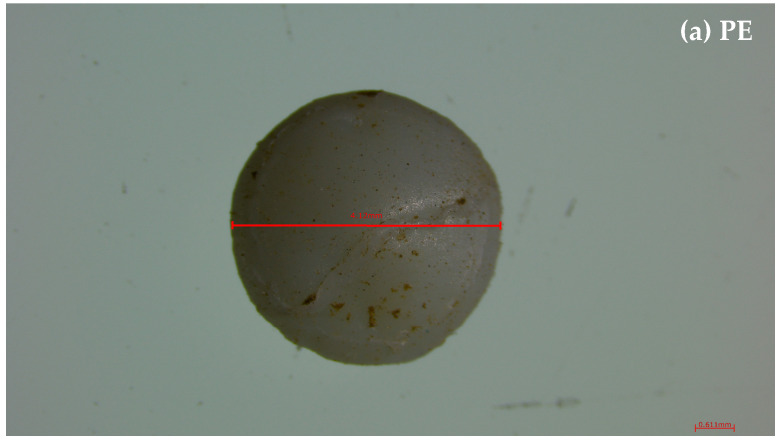

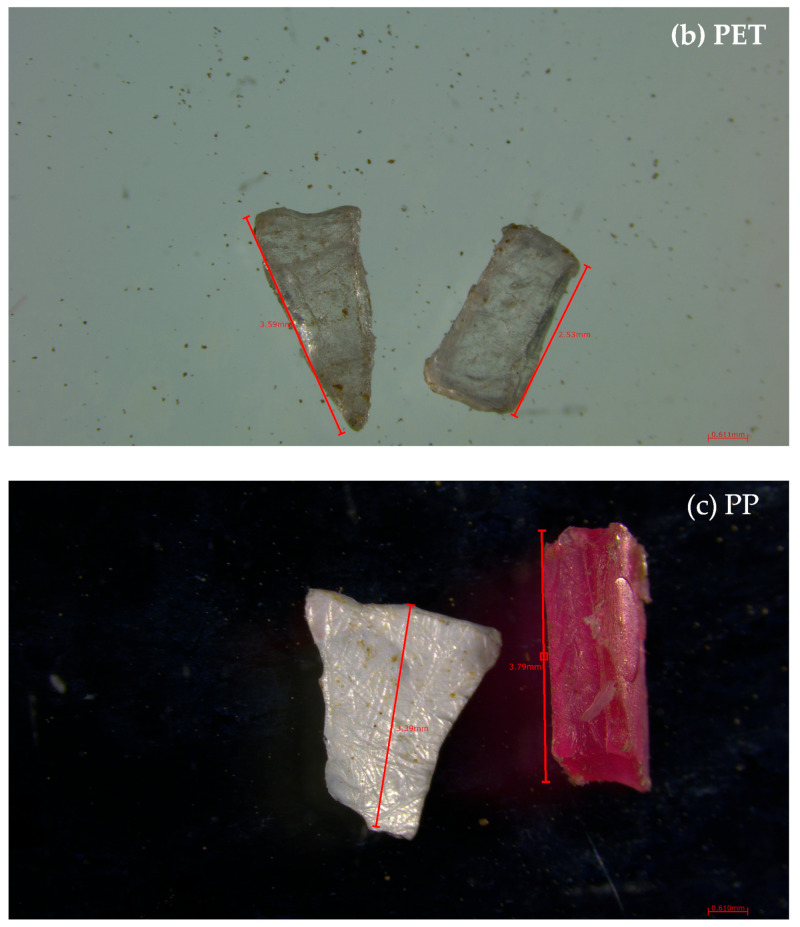
Size, color, and shape characteristics of microplastics used in this study: (**a**) polyethylene (PE), (**b**) polyethylene terephthalate (PET), and (**c**) polypropylene (PP).

Dried samples collected on the sieves were examined using a stereomicroscope (Leica S Apo, Leica Microsystems GmbH, Wetzlar, Germany) to visually identify and count microplastics (MPs) based on their physical characteristics (size, color, and shape) (Figure 1). Suspected particles within the size range of 0.1–5 mm were further analyzed via attenuated total reflectance Fourier-transform infrared (ATR-FT-IR) spectroscopy (Frontier, Perkin Elmer, Waltham, MA, USA) for chemical characterization. Only particles with a spectral match greater than 90% to reference compounds in the device’s library were identified and classified as microplastics.

### 2.4. Statistical Analysis

STATISTICA 12.0 software was used for all statistical analysis [38]. The effects of the incubation period and the microplastic amendment were assessed using two-way analysis of variance (ANOVA). Additionally, the impact of phosphorus processes was assessed using one-way ANOVA for every pollutant dose. Tukey’s HSD multiple comparison test was used for post hoc analysis in cases where significant effects were found (Table 2).

## 3. Results and Discussion

### 3.1. Total P

**Figure 2:**
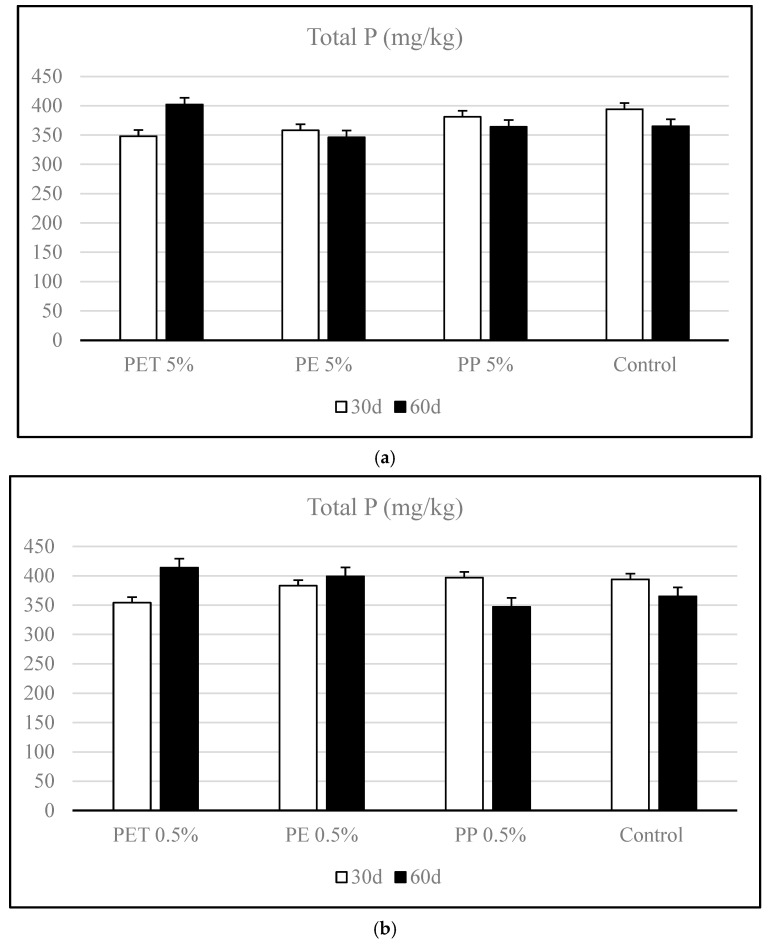
Variation of total phosphorus (TP) in soils contaminated with different types of microplastics (polyethylene [PE], polypropylene [PP], and polyethylene terephthalate [PET]) during the incubation period. (**a**) High-dose contamination (5% *w*/*w*), (**b**) Low-dose contamination (0.5% *w*/*w*).

The variation of total P in soils contaminated with microplastics during the incubation period is presented in Figure 2. Among the treatments, PET 5% and 0.5% showed a moderate increase (13.43% and 14.49%) in total phosphorus from day 30 to day 60, suggesting that PET microplastics may enhance phosphorus accumulation in the soil matrix over time (Figure 2a). This could be attributed to potential interactions between PET degradation products and soil colloids that reduce phosphorus mobility or facilitate its retention [10,39]. In contrast, PE 5% exhibited a slight decrease (3.35%) in total phosphorus over the same period. This trend might reflect enhanced phosphorus turnover or mineralization in PE-amended soils, possibly linked to microbial activity shifts or a decline in organic matter content due to the presence of PE [12,39].

Similarly, PP 5% contamination showed a moderate reduction (4.46%) in total phosphorus by day 60 compared to day 30, though the magnitude of change was less pronounced than in the PE treatment. This indicates that while PP may influence phosphorus dynamics, its effect on long-term phosphorus retention is limited [40].

In the PET 0.5% contamination, total phosphorus content increased markedly (17%) from day 30 to day 60, indicating a potential enhancement in phosphorus retention or accumulation (Figure 2b). This trend mirrors the pattern observed at the 5% PET dose, suggesting that PET may consistently promote phosphorus stabilization in the soil, even at lower concentrations [41].

PE 0.5% also exhibited a slight increase (4.17%) in total phosphorus during incubation. This response, differing from the reduction seen in the 5% PE treatment, shows that low concentrations of PE may be less disruptive to phosphorus availability and may even contribute to phosphorus conservation under certain conditions [42]. Conversely, PP 0.5% showed a clear decline (12.59%) in total phosphorus between day 30 and day 60. This trend suggests a potential enhancement of phosphorus mineralization or leaching in the presence of PP, possibly due to alterations in microbial activity or soil structure [39,40].

The ANOVA analysis revealed that the main effects of dose and incubation time on TP levels were statistically significant. Besides, the interaction between dose and time was determined to be statistically significant (*p* < 0.05), indicating that the effect of dose on total P varied depending on the incubation period. This suggests a time-dependent response, where the influence of microplastic doses on phosphorus dynamics changes over time. Therefore, when evaluating the impact of microplastics on phosphorus availability, both dose and exposure time should be considered jointly. This interaction may be associated with microbial transformation processes or the degradation behavior of different polymer types over time [41]. For all microplastic types, the interaction between dose and time was determined to be statistically significant (*p* < 0.05).

A comparative evaluation of 0.5% and 5% microplastic contamination revealed distinct dose-dependent effects on soil total P content across different polymer types. Total-P levels were found to be lower in soils exposed to high doses of microplastics. Among them, PET consistently increased total phosphorus content over time at both concentrations; however, the magnitude of increase was more pronounced at the 0.5% dose, suggesting that lower concentrations of PET may enhance phosphorus retention more efficiently, possibly due to moderate stimulation of microbial stabilization pathways without inducing toxic effects [42].

For PE, a contrasting trend was observed between doses. While the 5% PE contamination resulted in a decrease in total P over time—likely due to enhanced phosphorus mineralization or microbial imbalance—the 0.5% dose led to a slight increase in total P, indicating a more neutral or slightly beneficial effect at lower exposure levels. This highlights that high concentrations of PE may disrupt phosphorus cycling, while low levels may allow soil systems to maintain or even improve phosphorus conservation [12].

In the case of PP, both doses led to reductions in total phosphorus over time, but the decline was more substantial at 0.5%. This unexpected pattern could reflect accelerated mineralization or leaching processes driven by changes in soil microbial activity or a structure specific to PP, independent of concentration. Alternatively, it may suggest that PP’s impact is less dose-sensitive and more related to its physicochemical properties.

Overall, the comparison indicates that PET exhibits the most consistent and dose-sensitive positive effect on phosphorus retention, while PE shows clear dose-dependent divergence and PP tends to negatively influence phosphorus levels regardless of dose. These findings demonstrate the intricacy of the interactions between microplastics and soil and emphasize the significance of accounting for both concentration and polymer characteristics when evaluating the ecological effects of microplastic pollution on the dynamics of soil nutrients [40,43].

### 3.2. Available Phosphorus

**Figure 3:**
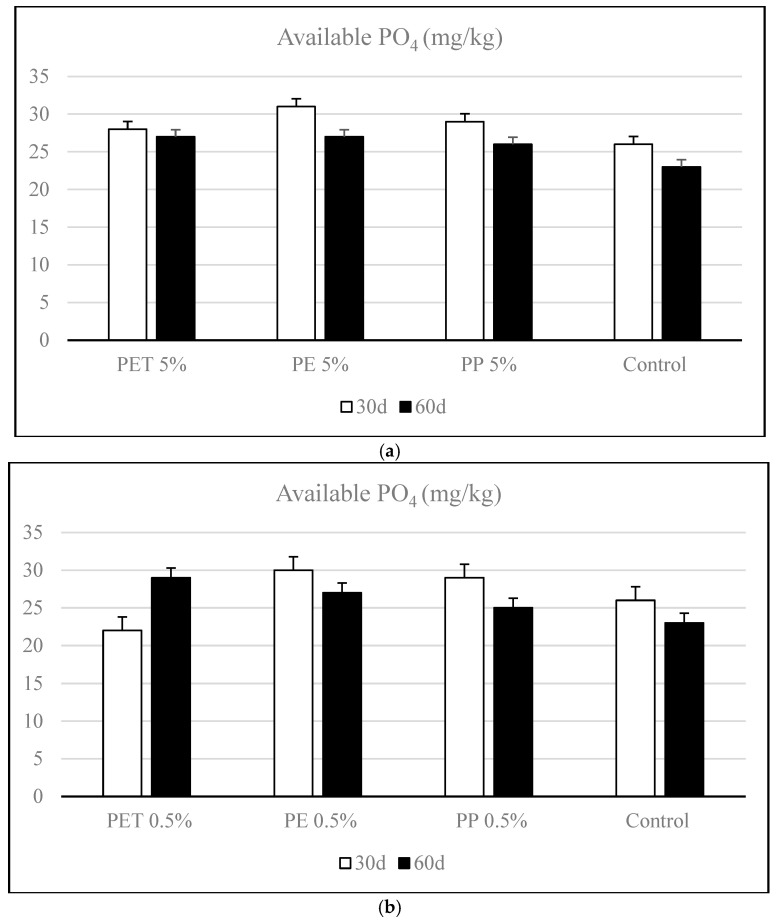
Variation of available phosphorus (AP) in soils contaminated with different types of microplastics (polyethylene [PE], polypropylene [PP], and polyethylene terephthalate [PET]) during the incubation period. (**a**) High-dose contamination (5% *w*/*w*), (**b**) Low-dose contamination (0.5% *w*/*w*).

Figure 3 illustrates the changes in available phosphorus levels during the incubation period in soils contaminated with different amounts of microplastics. The effects of 5% microplastic contamination on soil AP concentrations were evaluated, revealing a general decline in phosphorus availability over time (Figure 3a). This trend suggests a potential shift in phosphorus transformation dynamics or increased microbial immobilization during the incubation period [12,42].

Among the MP types, PE 5% initially exhibited the highest available phosphate content on day 30. However, by day 60, a moderate reduction (13%) was recorded, suggesting possible microbial uptake or conversion to less available forms. Similarly, PP 5% and PET 5% contamination also showed a decline (10.3% and 3.57%) in AP levels over time, although the magnitude of decrease was less pronounced in the PET contamination, indicating a relatively more stable phosphorus availability under PET influence. These findings suggest that while microplastics at 5% may initially increase or maintain available phosphate through enhanced mineralization or altered soil properties, their long-term effect tends toward decreased phosphate availability, likely due to shifts in microbial activity or phosphorus fixation [44]. The extent of this effect appears to be polymer-dependent, with PET showing the least impact on phosphorus depletion over time.

At the 0.5% dose, PET-contaminated soils showed a notable increase (31.8%) in AP levels from day 30 to day 60, suggesting enhanced phosphorus mineralization or reduced immobilization, potentially linked to microbial stimulation (Figure 3b). In contrast, at 5% PET, AP levels remained relatively stable or slightly declined (3.5%), indicating that higher PET concentrations may reduce the positive effect or introduce mild inhibitory conditions over time.

For PE, both 0.5% and 5% contamination resulted in a decrease (10% and 13%) in AP from day 30 to 60. However, the decline was less pronounced at 0.5%, implying that higher concentrations of PE may exert greater stress on microbial processes or increase phosphorus sorption and loss. While there was no significant difference between the doses, a statistically significant difference was detected when compared to the control soil (*p* < 0.05). Additionally, time had a statistically significant effect (*p* < 0.05), indicating that the temporal aspect also played a role in the observed changes.

PP contamination at both doses also showed a decrease in available phosphate over time, with the 0.5% dose maintaining slightly higher (14%) AP levels than the 5% treatment. However, there was no significant difference between the doses, although time had a statistically significant effect (*p* < 0.05).

The ANOVA results for AP indicated that both the dose of microplastics and incubation time had statistically significant effects (*p* < 0.05). These findings suggest that phosphorus availability in the soil is influenced independently by both the concentration of microplastics and the duration of exposure. According to the Tukey HSD test results, AP levels in the soil were significantly affected by the contaminant dose and incubation period for PET (*p* < 0.05); however, this interaction was not significant for PE and PP. Furthermore, the AP levels were not significantly influenced by the dose of PE or PP microplastics [45].

### 3.3. Alkaline Phosphatase Activity

**Figure 4:**
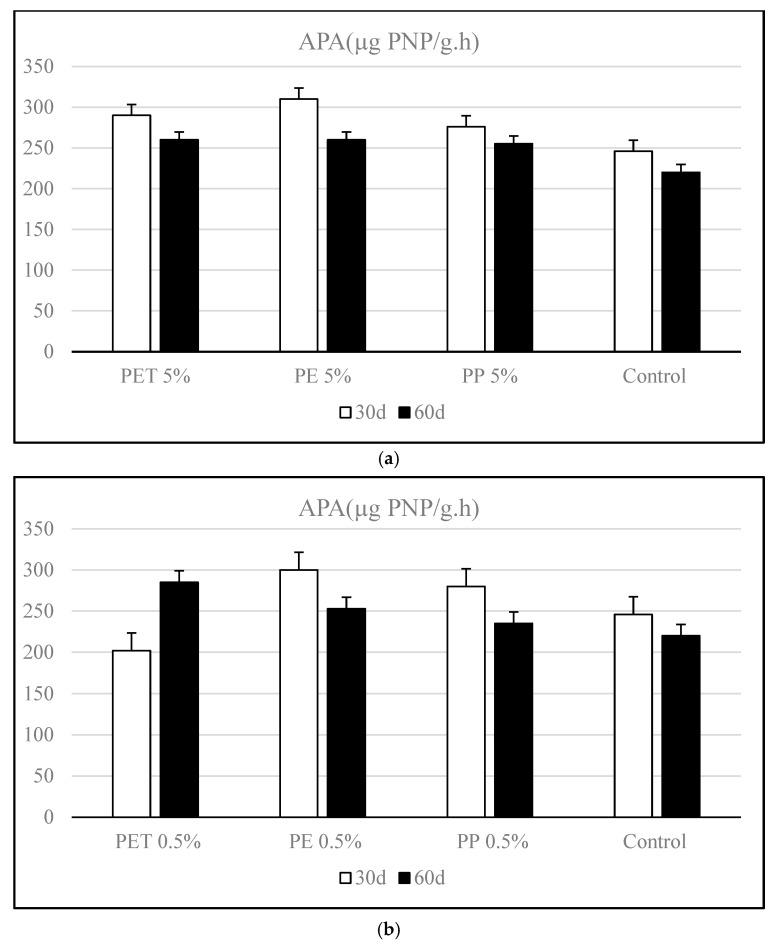
Variation of alkaline phosphatase activity (APA) in soils contaminated with different types of microplastics (polyethylene [PE], polypropylene [PP], and polyethylene terephthalate [PET]) during the incubation period. (**a**) High-dose contamination (5% *w*/*w*), (**b**) Low-dose contamination (0.5% *w*/*w*).

Figure 4 illustrates the changes in APA levels in microplastic-contaminated soils over the incubation period. Across all contamination, a general decreasing trend in APA was observed over time, indicating a decline in enzymatic activity as incubation progressed. Among the MP-treated groups, soils amended with 5% polyethylene (PE) exhibited the highest APA on day 30. However, by day 60, a moderate reduction (~16%) was recorded in this group. Similarly, the PET and PP contamination also showed elevated APA levels at day 30 compared to the control, but these declined by day 60 (Figure 4a).

APA varied significantly based on the type of microplastic applied and the incubation period. At a high microplastic dose (5%), APA levels increased by 1.43% to 43.5% after 30 days of incubation. However, by day 60, a decline (8.77%) was observed in PET-contaminated soils, whereas PE and PP treatments continued to show moderate increases, ranging from 2.76% to 8.5%. This suggests that the degradation products of PET accumulating in the soil over time may exert an inhibitory effect on APA.

Both PE and PP contamination showed a slight decrease (7.6–16%) in APA from day 30 to day 60. These declines suggest that although these microplastics may initially stimulate microbial activity, their influence diminishes over time—possibly due to slower degradation rates or reduced microbial stimulation—indicating that their effects are not sustained in the long term.

The ANOVA analysis showed that the incubation period and dose had statistically significant influences on APA levels. Additionally, the dose–time interaction was statistically significant (*p* < 0.05), suggesting that the incubation period affected the dose’s impact on APA.

A clear dose-dependent response was observed when comparing the effects of 0.5% and 5% microplastic contamination on soil APA (*p* < 0.05). The amount of APA in the soil was shown to increase as the percentage of microplastics increased. Among all polymers tested, PET exhibited the most distinct contrast between doses. At 0.5%, PET significantly enhanced APA during incubation, suggesting a stimulatory effect likely related to improved soil moisture retention or microbial support (Figure 4b). However, at 5%, PET initially stimulated APA, but this effect declined markedly by day 60, indicating potential microbial stress or enzymatic inhibition at higher concentrations. Similarly, PE and PP microplastics showed a temporal decline (16.13% and 7.60%, respectively) in APA at both concentrations.

The findings of this study highlight a clear dose-dependent effect of microplastics on soil APA, a key enzyme involved in phosphorus cycling. Notably, low dose (0.5%) microplastic amendments, particularly those containing PET, were found to stimulate APA over time, suggesting that moderate microplastic inputs may enhance microbial or enzymatic functions in the short term. This positive response may be attributed to improved soil aeration, increased water retention capacity, or indirect stimulation of microbial communities that support enzymatic activity [7,8]. These findings highlight the significance of accounting for both microplastic type and concentration when evaluating their environmental risks, as different polymers interact differently with soil matrices and microbial systems.

At the 0.5% dose, PET consistently enhanced both APA and AP levels over the incubation period, accompanied by total P enrichment. This indicates that low doses of PET microplastics may stimulate microbial activity and promote both phosphorus mineralization and retention, supporting plant-available phosphorus forms. In contrast, at 5% PET, APA declined, total P increased, and AP remained relatively stable, implying that while PET may still retain phosphorus chemically through the formation of functional groups (e.g., carboxyl and hydroxyl) that promote phosphorus adsorption onto microplastic surfaces or soil particles, the concurrent decline in APA suggests microbial stress or suppression of phosphatase activity, thereby limiting the biological transformation of phosphorus into its bioavailable forms [40]. The decrease in APA indicates a reduction in the biological transformation of phosphorus and its conversion into bioavailable forms, whereas the increase in total phosphorus suggests that phosphorus is accumulating in the system without being utilized or is being retained by microplastic particles. This situation may represent a complementary scenario of a “phosphorus trap,” in which phosphorus is present but inaccessible to biotic processes. PE showed clear dose-dependent divergence. At 0.5%, total P slightly increased, AP declined moderately, and APA showed a mild reduction, indicating a relatively balanced system with limited disturbance. Microplastics (MPs) have been shown to reduce the amount of phosphorus accessible in soil by directly affecting the number of microbial functional genes involved in the mineralization of organic phosphorus [46].

However, at 5% PE, both APA and total P decreased, and AP showed a more pronounced decline—suggesting that higher doses of PE suppress microbial phosphatase activity and phosphorus availability, potentially through physical barrier effects or toxic interactions with soil microbiota.

In soils contaminated with PP, TP levels remained largely unchanged for both low (0.5%) and high (5%) application rates. In contrast, increases in both AP and APA were observed at each concentration.

Exposure to MPs was found to increase phosphatase activity; however, no significant change in available P content was observed with increasing MP concentrations for PP and PE. A slight decrease (6.89%) in available P was only detected at high concentrations in the presence of PET. Similarly, Tong et al. [47] found that while the available P content decreased as MP concentrations increased, exposure to polyethylene (PE) MPs enhanced phosphatase activity.

Alterations in phosphatase activity following MP addition are not always sufficient to account for the observed changes in available phosphorus. The bioavailability of phosphorus is predominantly governed by microbial processes (organic acid secretion, enzyme production, microbial mineralization), including the mineralization of organic phosphorus and the solubilization of inorganic phosphate [26,42].

Previous studies have shown that MPs affect soil enzyme activity through several mechanisms. When MPs are introduced into the soil, they disrupt the structure of soil aggregates, leading to the release of previously protected organic matter. This released organic material serves as a substrate for microbial communities, thereby enhancing microbial activity [2,13]. In addition, due to their high adsorption capacity, MPs can act as microhabitats that promote bacterial colonization, which may alter the functional dynamics of soil ecosystems and further stimulate microbial proliferation [48,49].

MPs also have the capacity to modify the physicochemical properties and nutrient composition of the soil, directly influencing the efficiency and activity of extracellular soil enzymes [50].

### 3.4. Microplastic Identification and Degradation

**Figure 5:**
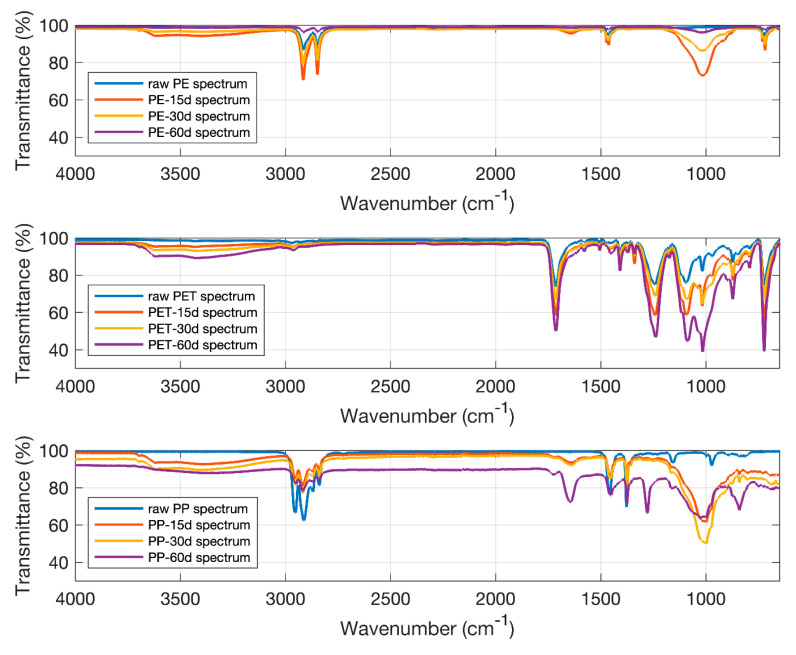
The FTIR spectra of the PE, PET and PP used in this study.

FTIR spectral analysis revealed clear evidence of chemical degradation in all microplastic types over the 60-day incubation period (Figure 5). For polyethylene (PE), the appearance of new absorption bands, particularly in the carbonyl (C=O) stretching region, indicates oxidative degradation characterized by polymer chain scission and the formation of oxygen-containing functional groups such as carbonyls. In the case of polyethylene terephthalate (PET), the spectral changes suggest both hydrolytic and photooxidative degradation, as evidenced by alterations in ester bond regions and the emergence of hydroxyl and carbonyl peaks. Similarly, polypropylene (PP) showed signs of oxidative degradation, with the development of carbonyl-related peaks and shifts in the fingerprint region.

**Figure 6:**
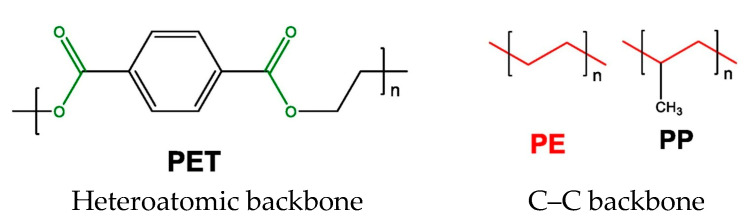
Chemical backbone structures of selected plastic polymers. C–C backbone: polyethylene (PE), polypropylene (PP). Heteroatomic backbone: polyethylene terephthalate (PET). Polymers with highly stable carbon–carbon (C–C) backbones are highlighted in red, whereas hydrolysable chemical bonds are highlighted in green.

Polymers such as PET possess a heteroatomic backbone, whereas PE and PP are characterized by a carbon-carbon (C-C) backbone (Figure 6). Compared to plastics containing hydrolysable bonds, those with a purely C-C backbone are more resistant to degradation [41].

Several studies have documented the gradual oxidation of PP in natural environments, with oxidation leading to the formation of carbonyl groups and eventual polymer chain scission [51]. However, PP’s degradation is still slower compared to PET, and like PE, it is relatively resistant to hydrolysis under typical environmental conditions [52]. The presence of carbonyl (C=O) peaks and broad O–H bands across all polymer types confirms that environmental conditions triggered chemical transformations in the plastic matrices. These changes reflect the breakdown of polymer structures and the introduction of polar functional groups, which are indicative of advanced degradation processes. Among the tested polymers, PET exhibited the most pronounced and rapid signs of degradation, likely due to its susceptibility to hydrolysis and photodegradation mechanisms. Overall, the FTIR spectra provide strong evidence that all microplastics underwent oxidative and/or hydrolytic degradation during the experimental period, with PET being the most significantly altered. Studies have shown that PET degrades faster than PE and PP under similar environmental conditions due to these more reactive chemical linkages [53]. Hydrolytic degradation of PET is particularly prevalent in moist conditions, where water molecules can break the ester bonds, leading to the formation of shorter polymer chains and the release of degradation byproducts such as terephthalic acid [54].

In terms of time-dependent degradation behavior, PET exhibited the most rapid and complex structural changes, indicating high susceptibility to environmental factors such as hydrolysis and photooxidation. PP showed moderate levels of oxidative degradation, with the formation of carbonyl-containing groups and noticeable changes in the polymer backbone. In contrast, PE underwent the least structural alteration, demonstrating the highest resistance to degradation among the tested polymers. These results suggest that the degradation rates and mechanisms are strongly influenced by the intrinsic chemical structure of each polymer, with PET being the most degradable and PE the most persistent under the given soil conditions.

Polyethylene (PE) is composed of a saturated hydrocarbon backbone consisting solely of repeating –CH_2_–CH_2_– units, which imparts a high degree of chemical stability. Due to the absence of reactive functional groups, PE is inherently resistant to common degradation mechanisms such as hydrolysis and photooxidation. In natural environments, where such energy inputs are limited, the radical initiation step is inefficient, resulting in a considerably slower degradation rate. Zhang et al. [55] reported that PE, due to its saturated nature, does not degrade significantly even over extended periods of environmental exposure. Consequently, PE remains one of the most persistent and degradation-resistant polymers under typical environmental conditions [56,57].

As a result of these degradation processes, it is assumed that functional groups such as carboxyl, carbonyl, and hydroxyl, accompanied by low-molecular-weight compounds, are released into the soil matrix [58,59]. These substances can influence microbial activity in the soil, thereby shaping the chemical speciation and bioavailability of phosphorus. In particular, the functional groups released from degraded PET and PP may enhance complexation with phosphate ions or facilitate the transformation of phosphorus from more stable inorganic forms into more mobile and bioavailable species.

In soils amended with PET at 0.5%, alkaline phosphatase activity (APA) significantly increased over the 60-day incubation period. Consistent with these processes, the results indicate that available phosphorus levels increased in soils contaminated with PET by the end of the incubation period. This suggests that the degradation products of PET may have stimulated microbial activity, particularly those involved in phosphorus mineralization. The enhanced APA is likely associated with increased microbial secretion of phosphatases or organic acids that facilitate phosphorus solubilization. These findings align with the hypothesis that PET, due to its higher susceptibility to hydrolytic and photooxidative degradation, releases bioactive compounds that can improve phosphorus bioavailability in soil.

In contrast, APA levels gradually declined over time in soils treated with PE (~16%) and PP (7–16%). This reduction may be attributed to the chemical inertness of these polymers, which degrade more slowly and release fewer functional degradation products. The saturated hydrocarbon backbone of PE and the hydrophobic nature of PP likely limit their interaction with soil microbial communities, resulting in minimal stimulation or even suppression of enzymatic activity.

## 4. Conclusions

This study provides compelling evidence that microplastics significantly influence soil phosphorus dynamics in a manner that is both polymer-specific and dose-dependent. In soils contaminated with MPs, AP and APA levels increased relative to the control. In highly contaminated soils (5%), an increase of approximately 7–16% was recorded after 30 days of incubation, and by the end of the incubation period, an increase of 11.5–14.8% was noted. In soils contaminated with a low dose (0.5%), AP increased by 10.3–13% after 30 days and by 8–26.7% following 60 days of incubation. Similarly, a high microplastic concentration (5%) led to an increase in APA levels ranging from 1.43% to 43.5% after 30 days of incubation. However, by day 60, a decline (8.77%) was observed in PET-contaminated soils, whereas soils with PE and PP treatments continued to show moderate increases, ranging from 2.76% to 8.5%.

Microplastic degradation patterns also varied across polymer types, with PET undergoing the most pronounced chemical alterations due to hydrolytic and photooxidative processes. PP exhibited moderate degradation, while PE showed the highest resistance to environmental breakdown, consistent with its chemically saturated and UV-resistant structure. PET, which undergoes notable degradation via hydrolysis and photo-oxidation, releases functional groups (e.g., carboxyl, hydroxyl) that may enhance phosphorus bioavailability by facilitating its transformation into more mobile forms. In contrast, PE and PP, due to their hydrophobic and inert nature, degrade minimally.

This study showed that microplastic dose and incubation time affect soil phosphorus dynamics in different ways. These findings indicate that available P is more sensitive to microplastic exposure and highlight the need to consider both dose and exposure time when assessing nutrient availability under microplastic contamination.

## Figures and Tables

**Table 1:** General properties of the soil utilized in this research [24].

Parameters	Values
pH (1:5)	7.67
EC, µS/cm 25 °C (1:5)	210
Total N, %	0.22
Total P, %	0.08
NH_4_^+^-N, mg·kg^−1^	8.74
NO_3_^−^-N, mg·kg^−1^	24.6
Organic matter, %	3.42

**Table 2:** Results of ANOVA for the analysis of the main effects of contaminant doses and incubation times for PE, PP and PET.

	PE				PP				PET			
Sources of Variation	df	MS	F	*p*	df	MS	F	*p*	df	MS	F	*p*
Dependent Variable: Total-P												
Contaminant dose	2	2644	337.6	*p* < 0.001	2	85	27.2	*p* < 0.001	2	157	51.3	*p* < 0.001
Time	1	450	57.4	*p* < 0.001	1	4418	1420.1	*p* < 0.001	1	3254	1064.8	*p* < 0.001
Contaminant dose × time	2	962	122.8	*p* < 0.001	2	473	151.9	*p* < 0.001	2	3696	1209.5	*p* < 0.001
Error	12	8			12	3			12	3		
Dependent Variable: Available-P												
Contaminant dose	2	44.22	88.44	*p* < 0.001	2	15.5	46.5	*p* < 0.001	2	9.39	33.8	*p* < 0.001
Time	1	68.06	136.11	*p* < 0.001	1	50	150	*p* < 0.001	1	5.56	20	*p* < 0.001
Contaminant dose × time	2	1.56	3.11	0.08156	2	0.5	1.5	0.262144	2	40.06	144.2	*p* < 0.001
Error	12	0.5			12	0.33			12	0.28		
Dependent Variable: APA												
Contaminant dose	2	4715	424.3	*p* < 0.001	2	1541	210.1	*p* < 0.001	2	3187	651.8	*p* < 0.001
Time	1	8065	725.8	*p* < 0.001	1	4802	654.8	*p* < 0.001	1	280	57.3	*p* < 0.001
Contaminant dose × time	2	314	28.3	*p* < 0.001	2	277	37.8	*p* < 0.001	2	6188	1265.8	*p* < 0.001
Error	12	11			12	7			12	5		

## Data Availability

The data presented in this study are available on request from the corresponding author.

## Abbreviations

The following abbreviations are used in this manuscript:

Total P	Total Phosphorus
AP	Available Phosphorus
APA	Alkaline Phosphatase Activity
PET	Polyethylene Terephthalate
PE	Polyethylene
PP	Polypropylene
MPs	Microplastics
PS	Polystyrene
PVC	Polyvinyl chloride
WHC	Water Holding Capacity
PTFE	Polytetrafluoroethylene
PSB	Phosphate-solubilizing Bacteria
